# Construction of chicken infectious anemia virus infectious clone and study on its pathogenicity

**DOI:** 10.3389/fmicb.2022.1016784

**Published:** 2022-09-23

**Authors:** Jinjin Wang, Yan Li, Yawen Zhang, Longfei Chen, Lichun Fang, Shuang Chang, Yixin Wang, Peng Zhao

**Affiliations:** ^1^College of Animal Science and Veterinary Medicine, Shandong Agricultural University, Tai’an, China; ^2^Shandong Academy of Agricultural Sciences, Jinan, China

**Keywords:** chicken infectious anemia virus, pathogenicity, VP3 fusion protein, fowl pox virus, infectious clone

## Abstract

Chicken infectious anemia virus (CIAV) can be transmitted through contaminated live poultry vaccine. However, the pathogenicity of contaminated CIAV strains is rarely reported. Previously, the chickens showed the typical symptoms of anemia after using the attenuated live fowl pox virus (FPV) vaccine. Therefore, exogenous CIAV contamination was suspected. We detected anti-CIAV antibodies in SPF chicks vaccinated with the FPV vaccine. CIAV contamination was confirmed in the FPV vaccine, and the CIAV strain was named JS2020-FPV. This study aims to rescue JS2020-FPV by reverse genetic assays and investigate its pathogenicity. Firstly, double-copies infectious clone of JS2020-FPV was constructed. For the pathogenicity study, infectious clone of JS2020-FPV was used to inoculate 1-day-old SPF chicks. The typical symptoms of anemia were observed in the JS2020-PFV group 14  days post inoculation. The hematocrit and body weight of chicks in the JS2020-PFV group were significantly lower than those in the mock group. Notably, the thymus development index and antibody levels of NDV were lower in chicks in the JS2020-PFV group than those in the mock group. Different degrees of apoptosis of MSB1 and DF-1 were observed after inoculated with the JS2020-FPV VP3 recombinant fusion protein expressed by *E. coli* system, indicating that VP3 induced apoptosis in the transformed cells. Overall, the pathogenicity of the CIAV detected in the contaminated vaccine was confirmed by inoculating SPF chicks with the double-copies infectious DNA clone in this study. Our findings indicate that the dangers of vaccine contamination cannot be ignored.

## Introduction

Chicken infectious anemia is an immunosuppressive disease caused by the chicken infectious anemia virus (CIAV), which belongs to the family *circoviridae* (Noteborn et al., 1991). *In vivo*, CIAV targets thymic precursor T cells and bone marrow hematopoietic stem cells (Adair, 2000). When infected with CIAV, symptoms of thymic atrophy and typical anemia, such as decreased hematocrit (HCT), could be seen in infected chickens. This can cause a serious economic loss to the breeding industry.

The use of live vaccines contaminated by CIAV is one of the transmission routes of CIAV. Although several cases of CIAV contamination in vaccines have been reported, the pathogenicity of contaminated CIAV has not been systematically reported. On the one hand, most tests used molecular methods and viable CIAV is not isolated. On the other hand, it is difficult to culture CIAV *in vitro*. Constructing the infectious clone is undoubtedly one of the best ways to evaluate its pathogenicity.

The contaminated fowl pox virus (FPV) vaccine was used in a farm of yellow broilers in Jiangsu province. After using the vaccine, typical symptoms of CIAV infection appeared in the chickens. Thus, CIAV contamination was suspected. In this study, the pathogenicity of the CIAV contaminated strain was observed by constructing double-copies infectious clone and using to inoculate chicks.

Homologous recombination was used first to construct the double-copies infectious clone of the CIAV contaminated strain. The advantage of this technology is that it can directly obtain live viruses by inoculating with the plasmid *in *vivo**. To confirm that this double-copies infectious clone plasmid can cause effective infection *in *vivo**, the anti-CIAV antibody was tested, and fluorescence quantitative PCR technology was used to detect CIAV nucleic acid in different samples, such as the cloaca and plasma. The results revealed that the time when it tested positive varied across samples. CIAV nucleic acid in the cloaca, plasma, liver, and spleen samples could be detected at 3 d.p.i., whereas it could be detected in the feather sac at 8 d.p.i., suggesting that viral shedding is relative early in the cloaca. The anti-CIAV antibodies could be detected in the serum of some chicks at 8 d.p.i. Serum antibodies of all test chicks were positive against CIAV at 11 d.p.i. These findings indicate that CIAV infection occurred after inoculation with the JS2020-PFV double-copies infectious cDNA. At 40 d.p.i., the chicks were euthanized and dissected. The fluid in the liver and spleen were grinded was used to inoculate MDCC-MSB1 cells. This provided a new technical approach to evaluate the infection and pathogenicity of CIAV. Lastly, apoptotic detection was performed using recombinant VP3 protein expressed by *E. coli* system on DF-1 and MSB-1 cells.

## Materials and methods

### Sample source and collection

A total of 28 one-day-old SPF chicks purchased from Jinan SAIS Poultry Co., LTD were randomly divided into the experience group and mock group. Each group had 14 chicks. Vaccine samples were filtered with a 0.22-μm filter, and 150 μL of the filtrate was injected per chick intramuscularly. Venous blood was collected at 14 days post inoculation (d.p.i.). The anti-CIAV antibody was detected using the avian CIAV antibody detection kit (IDEXX, United States). To further determine whether the virus contamination was exogenous, antibodies against avian leukosis virus subgroup J (ALV-J, IDEXX, United States), avian leukosis virus subgroup A (ALV-A, IDEXX, United States), reticuloendotheliosis virus (REV, IDEXX, United States), and fowl adenovirus (FAdV, Biocheck, Netherlands) were used. At 21 d.p.i., the animals were euthanized and their livers and spleens were stored at −80°C.

### Detection of CIAV and genomic sequencing

DNA from the liver and spleen was extracted. The whole genome of the CIAV genome was amplified using three primer pairs refer to the paper published from our laboratory (Fang et al., 2015; Table 1). Specific bands revealed by gel electrophoresis were recovered using a commercial kit (OMEGA). The product was ligated into the pMD-18 T vector, and sequenced by Shanghai Biotechnology Technology Co.

**Table 1:** Primers used for CIAV amplification and detection.

Primers	Sequence(5′→3′)	Product length
PF1	TGACGTATTCCGAGTGGTTACTATTCCATCACCATT	
PR1	TGACGTGATTGTGCGATTAAGCCATTTGCTGCGTTT	
PF2	TGACGTGGATCCTTCAGCCCCGTGGCGAGTCTTCTC	
PR2	TGACGTGGATCCCTCATTCTTAGTGGCAAGGAGCTG	
CAV-F1	GCATTCCGAGTGGTTACTATTCC	842 bp
CAV-R1	CGTCTTGCCATCTTACAGTCTTA	
CAV-F2	CGAGTACAGGGTAAGCGAGCTAAA	990 bp
CAV--R2	TGCTATTCATGCAGCGGACTT	
CAV-F3	ACGAGCAACAGTACCCTGCTA	802 bp
CAV-R3	CTGTACATGCTCCACTCGTT	
C-F	GCATTCCGAGTGGTTACTATTCC	843
C-R	CGTCTTGCCATCTTACAGTCTTAT	
CC-F	TACGTCACAGCCAATCAGAA	418
CC-R	GCATTGCAGATCTTAGCGT	

Products amplified by CAV-F1/R1, CAV-F2/R2 and CAV-F3/R3 overlap with each other. C-F/R is outer primers for Nested-PCR amplification, CC-F/R is inner primers for Nested-PCR amplification.

### Construction of JS2020-PFV infectious clone

**Figure 1:**
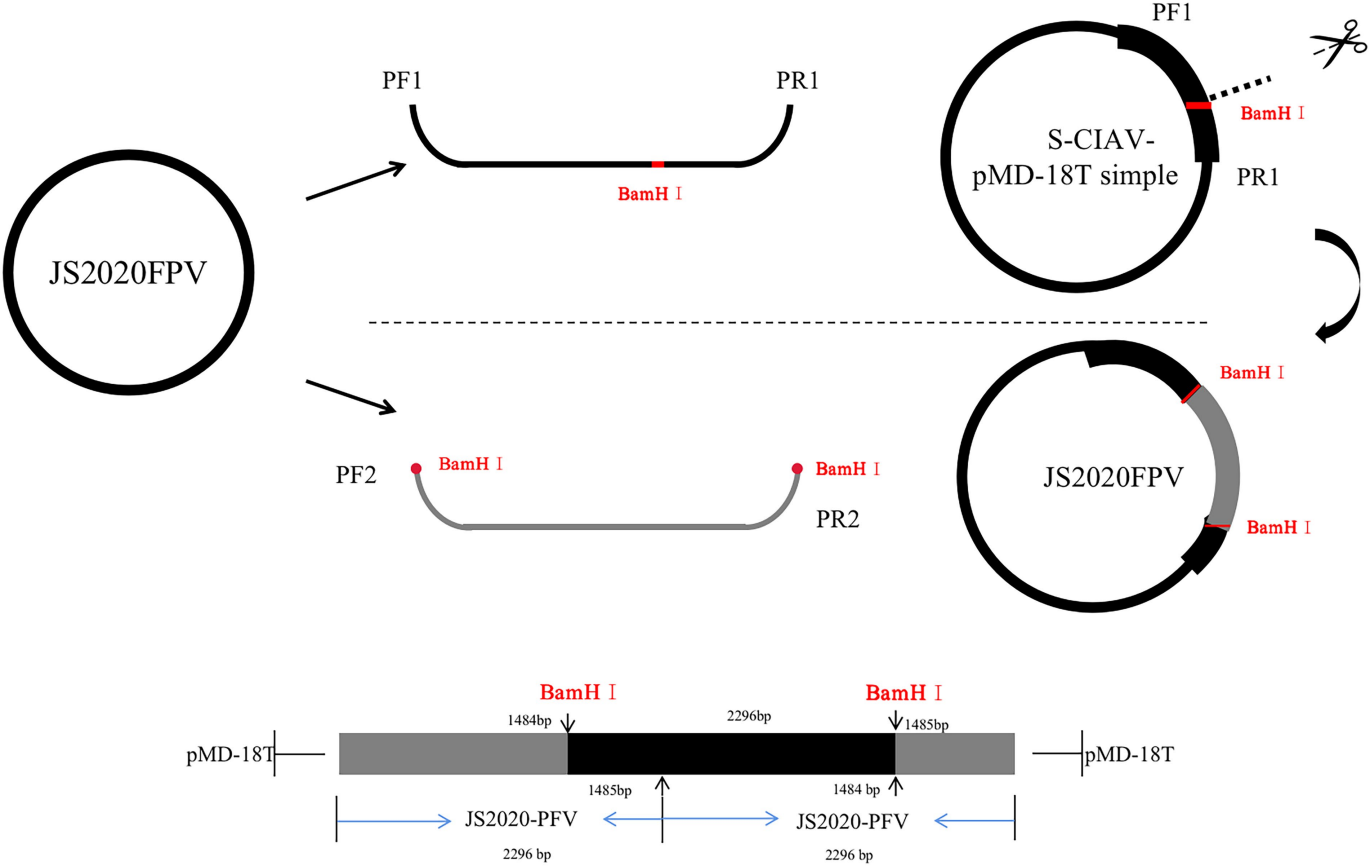
Schematic showing construction of the JS2020-PFV double-copies infectious clone. Primers PF1/R1 were used to amplify the single-copy clone. S-JS2020-PFV was digested with the *BamH* I endonuclease. The amplification product of PF2/R2 was inserted into the gap through homologous recombination to construct the JS2020-PFV double-copies infectious clone.

Two rounds of amplification were performed to synthesize the whole gene sequence of JS2020-PFV using primers PF/R1 and PF/R2. The amplification product obtained using primer pair PF1/PR1 was ligated with the pMD-18 T vector to build S-JS2020-PFV, a single-copy infectious clone. S-JS2020-PFV was digested using the *BamH* I endonuclease, the amplification product of PF2/R2 was inserted into the gap through homologous recombination to construct the JS2020-PFV double-copies infectious clone (Figure 1).

### Validation of infectivity of JS2020-PFV infectious clone *in vivo*

A total of 60 1-day-old SPF chicks were divided into two groups and raised under negative pressure air-filtration isolator. The double-copies infectious clone was used to inoculate 30 one-day-old SPF chicks (100 μL per chick) in the JS2020-PFV group. The 30 SPF chicks in the mock group were injected with PBS. The anti-CIAV antibody was detected using the IDEXX ELISA kit. At 40 d.p.i., the liver and spleen samples of all chicks were collected and DNA was extracted using a DNA extraction kit (TIANGEN, China). CIAV nucleic acid was amplified using nested-PCR according to the method established by our laboratory (Li et al., 2017a,b). The primers are shown in Table 1. The tissue-grinding fluid in which the nucleic acid was detected positive against CIAV was inoculated into MSB1 cells for isolating the virus. A murine antibody against CIAV-VP3 protein was used as the primary antibody, and FITC-labeled goat antimouse IgG was used as the secondary antibody for IFA.

**Table 2:** Detection of antibodies against CIAV.

group	The positive rate of antibody against CIAV at different time points (d.p.i.)						
	8	11	14	21	28	35	40
PBS	0	0	0	0	0	0	0
JS2020-PFV	23%	100%	100%	100%	100%	100%	100%

The proportion of SPF chicks with positive antibody against CIAV was the result of positive number divided by the total number.

### Investigation of pathogenicity of rescued JS2020-PFV

The probability of survival of all test chicks was evaluated regularly. At 1, 3, 5, 8, 11, 14, 21, 35, and 40 d.p.i., we measured the body weight. At 8, 14, 21, and 40 d.p.i., the anticoagulant was collected for HCT measurement. At 8, 14, 21, and 40 d.p.i., four chicks from each group were sacrificed and the immune organ indices were calculated with formula: organ weights (g) / body weight (g) × 100% × 100. To further evaluate the level of immuno suppression in chicks after CIAV inoculation, all chicks in the test group were vaccinated with the live attenuated NDV vaccine at 17 d.p.i. Serum was collected at 22 d.p.i. to analyze the antibody titer against NDV by performing HI.

### Analysis of the apoptotic characteristic of VP3

To analyze the apoptotic characteristics of VP3 of JS2020-PFV, we used the primers VP3FB (5′-CGGGATCCTTT CAAATGAACGCTCCCCAA-3′) and VP3RX (5′-CCCTCG AGGCCATCTTACAGTCTTATACA-3′) to amplify the VP3 of JS2020-PFV. A *BamH* I endonuclease restriction site is present in VP3FB and an *Xho* I endonuclease restriction site is present in VP3RX. The recombinant plasmid was constructed after ligating the product into the pET-32a vector. A total of 19 amino acids in front of JS2020-PFV VP1 were amplified using the homologous recombination primers, TYCVP1-32-3F (5′-GCCATGGCTGATATCGGATCCTGTAAGATGGCAAGACGAGCTCGC-3′) and TYCVP1-32-3R (5′-TCATTTGAAAGGATCCGCCACCGTCC TCTTCTGAA GGC-3′). The *BamH* I site was added upstream and downstream. The linearized pET-32a plasmid was ligated to the VP3 sequence using *BamH* I. Then the VP3 protein expression sequence was ligated to the CVP1 amplification product using homologous recombination. Thus, pET-32a-VP3-CVP1 was constructed in which these two inserted sequences share a start codon. After being analyzed electrophoretically, the expressed protein was purified using Ni-NTA chromatography and quantified using the BCA method. Then CEF, DF1, and MDCC-MSB1 cells were inoculated with the quantified protein to evaluate the degree of apoptosis.

## Results

### Identification of CIAV contamination

Results from antibody detection analysis revealed that all test chicks tested negative against the suspected pathogens, excluding CIAV, indicating that the vaccine was contaminated with CIAV. The full-length genome sequence of CIAV was amplified in the liver and spleen DNAs by PCR, and the CIAV strain was named JS2020-PFV (NCBI accession number: MW234428).

### Genome analysis of CIAV VP1 and construction of its infectious clone

**Figure 2:**
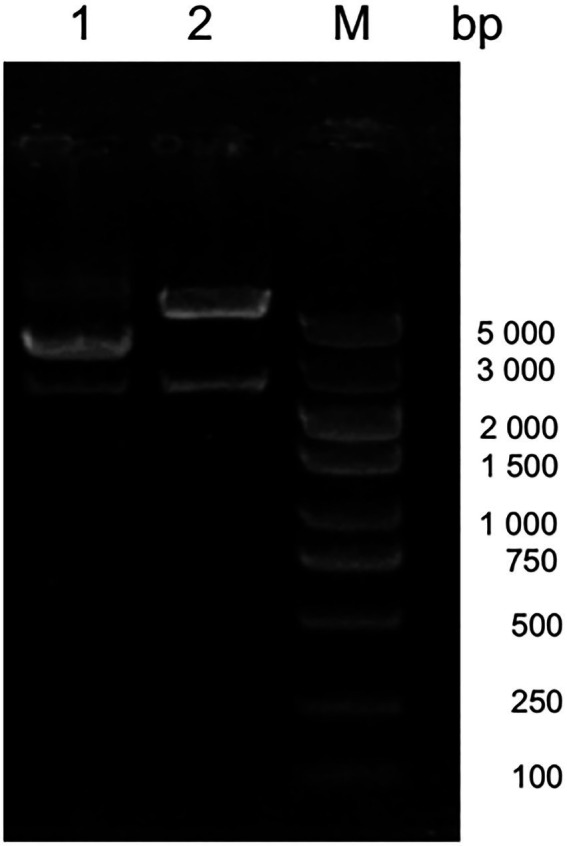
Digestion of the infectious clone plasmid. M 5000 DNA Marker, #1 original double-copies infectious clone plasmid, #2 digestion of the double-copies infectious clone.

Amino acid alignment of VP1 showed that JS2020-PFV was highly pathogenic at the key amino acid sites, which include 75 (V), 89 (K), 125 (I), 139 (K), 141 (Q), 144 (G), 157 (M), and 394 (Q). Target fragments were obtained in accordance with expected size after digesting the constructed double-copies plasmids using *Xcm* I (Figure 2). The double-copies infectious clone was quantified as 349.344 ng/L.

### Clinical manifestations of test chicks

**Figure 3:**
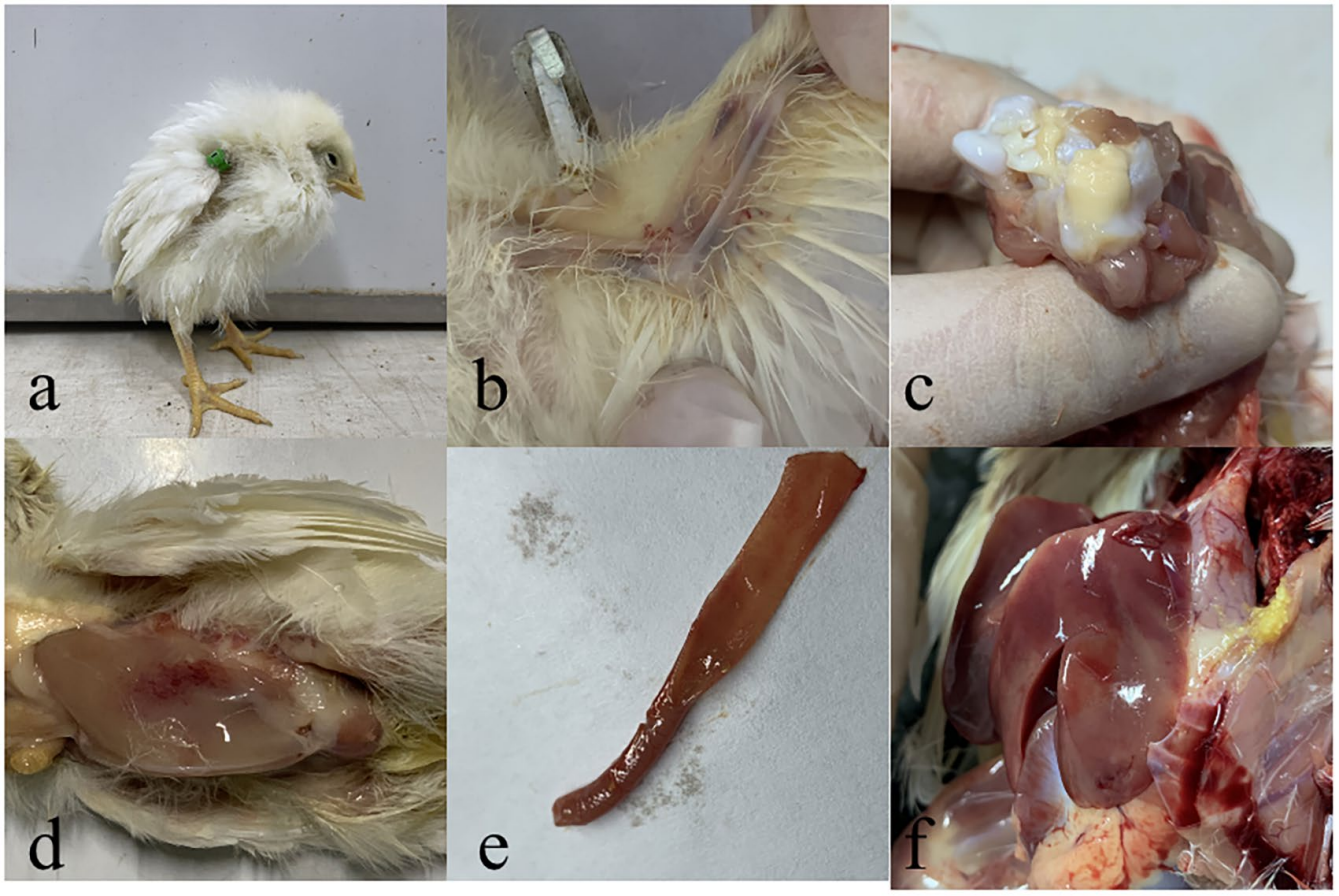
Typical clinical and pathological dissection symptoms of vaccinated chickens. **(a)** Chickens in in the JS2020-PFV group expressed depressed. **(b)** Their underwing showed bleeding, which manifested as blue wings. **(c)** The bone marrow was fatty. **(d)** The pectoral muscle showed bleeding. **(e)** The gut showed slight bleeding. **(f)** The liver had ischemia foci on the surface.

**Figure 4:**
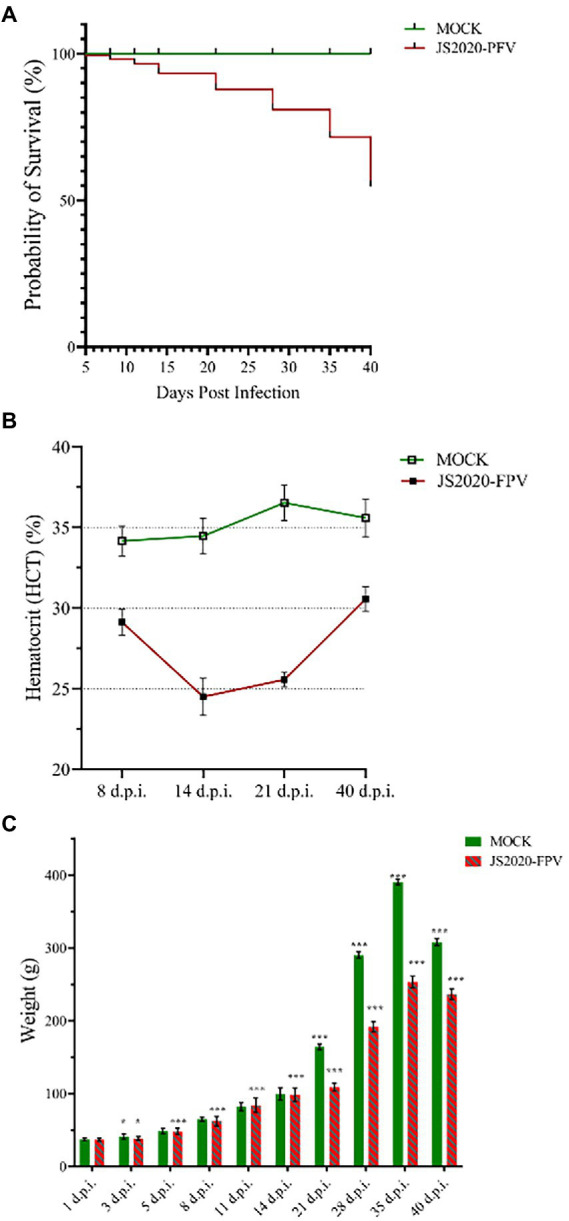
Clinical manifestations of test chicks. **(A)** Probability of survival, 23.3% mortality rate were recorded in the JS2020-PFV group (excluding normal dissection). No death occurred in the mock group. **(B)** Results of hematocrit (HCT; *n* = 4/group). The HCT of SPF chicks in the JS2020-PFV group was consistently lower than that of chicks in the mock group. In the JS2020-PFV group, it was lower than 27% from 14 d.p.i to 21 d.p.i. **(C)** Weight detection of experienced chicks showed average ± SD. Significant differences throughout the experiment are indicated by different labels next to the legend of each graph, *p < 0.05, ***p < 0.001.

One-day-old SPF chicks in the JS2020-PFV group were inoculated with 100 μL of double-copies of infected clones, which represents 4 × 10^11^ copies. Symptoms of mental depression and anorexia were observed in the JS2020-PFV group at 14 d.p.i. Some chicks showed typical underwing hemorrhaging. Moreover, symptoms of fatty marrow, intestinal bleeding, chest muscle bleeding, and liver anemia were observed in the JS2020-PFV group (Figure 3). The death of chicks was recorded in the JS2020-PFV group at 3 d.p.i. (Figure 4A). Death was constantly recorded in the test chick group, and a 23.3% mortality rate was recorded in the JS2020-PFV group (excluding normal dissection), whereas no deaths occurred in the mock group. From 8 d.p.i. the HCT of SPF chicks in the JS2020-PFV group was consistently lower than that of the mock group (Figure 4B). SPF chicks in the JS2020-PFV group gained significantly lesser weight than those in the mock group from 21 d.p.i. The trend chart visually displayed the weight change. The weight gain in the JS2020-PFV group was inhibited from 21 d.p.i. (Figure 4C).

### Immunosuppressive effect of JS2020-PFV

**Figure 5:**
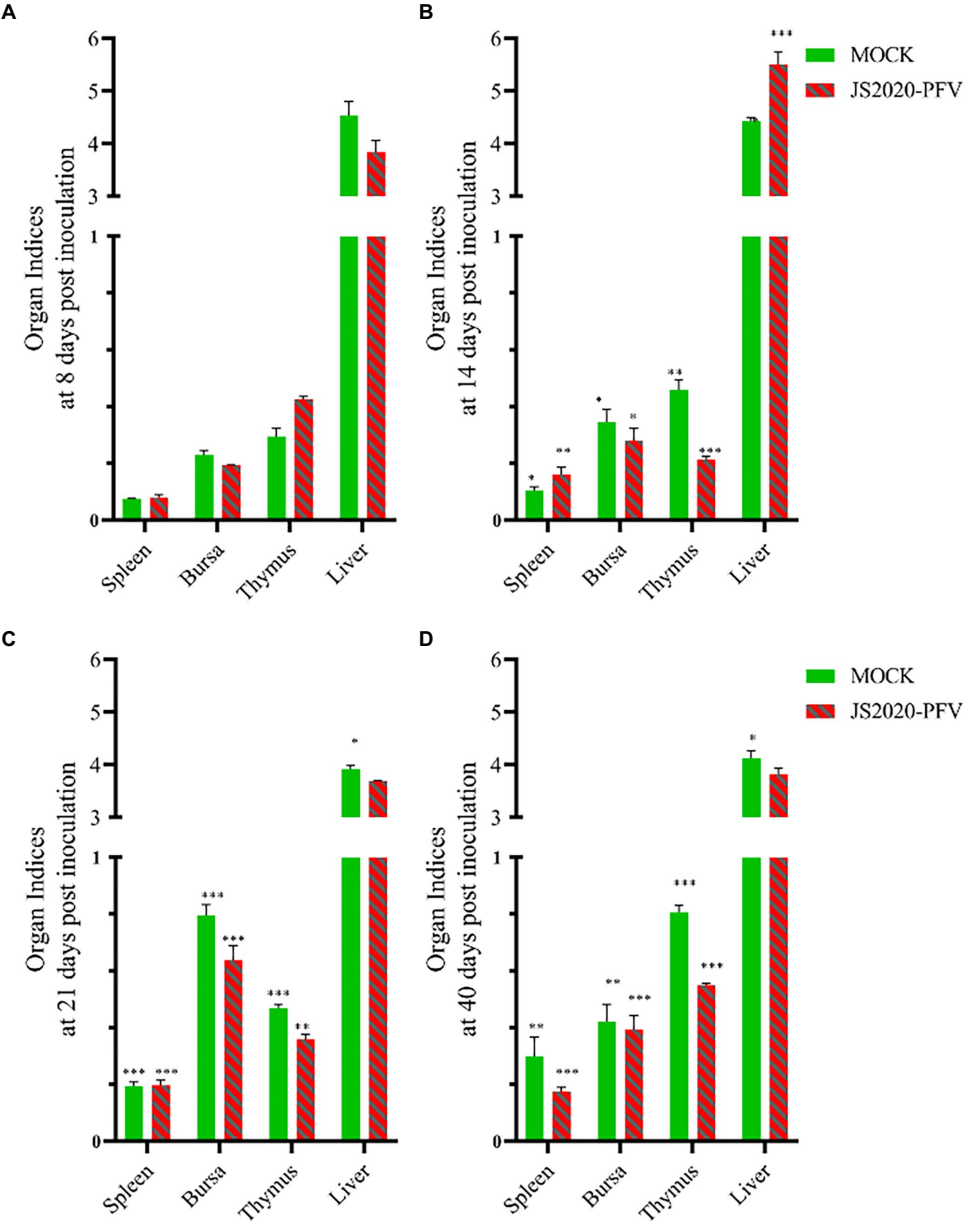
Organ indices at different days post inoculation (d.p.i.; *n* = 4/group). Organ index = immune organ weights (g) / body weight (g) × 100% × 100, **(A)** Organ indices at 8 d.p.i. **(B)** Organ indices at 14 d.p.i. **(C)** Organ indices at 21 d.p.i. **(D)** Organ indices at 40 d.p.i. *p < 0.05, **p < 0.01, ***p < 0.001.

**Figure 6:**
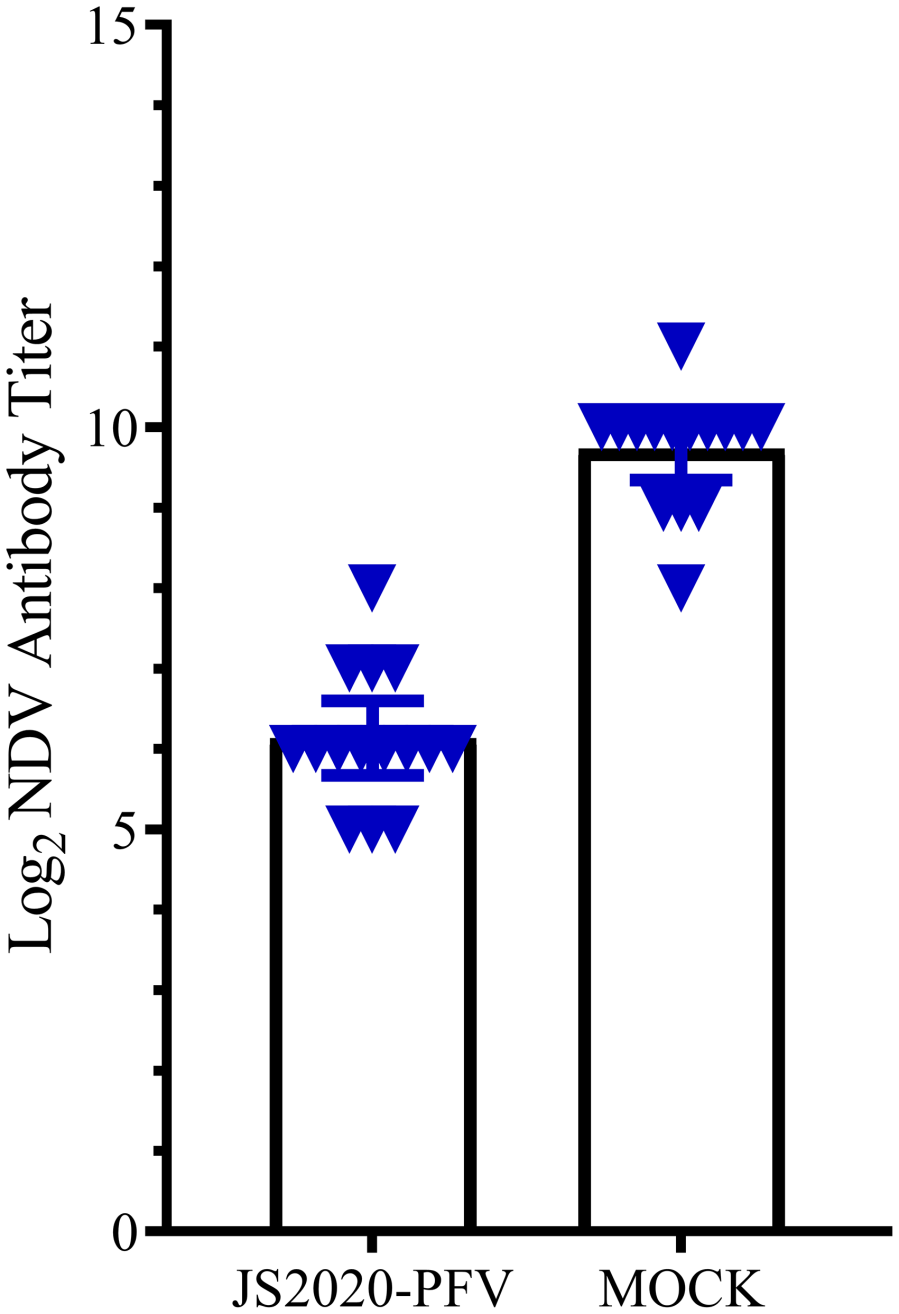
The NDV antibody titer in the experience group (*n* = 15/group). All chicks in the experience group were vaccinated with the NDV attenuated live vaccine at 17 d.p.i. The antibody titer against NDV was tested at 22 d.p.i. The NDV antibody titer was 6.3 in the JS2020-PFV group and 10.1 in the mock group (*p* < 0.05).

A comprehensive observation of various organs, such as the thymus, bursa fabricius, liver, and spleen (Figure 5), showed that in the JS2020-PFV group, the development index of the thymus and bursa was significantly lower than that in the mock group. The testing of NDV immunosuppression showed that the NDV antibody titer was 6.3 in the JS2020-PFV group and 10.1 in the mock group (Figure 6; *p* < 0.05).

### Organ damage and cytokine expression in infected chicks

**Figure 7:**
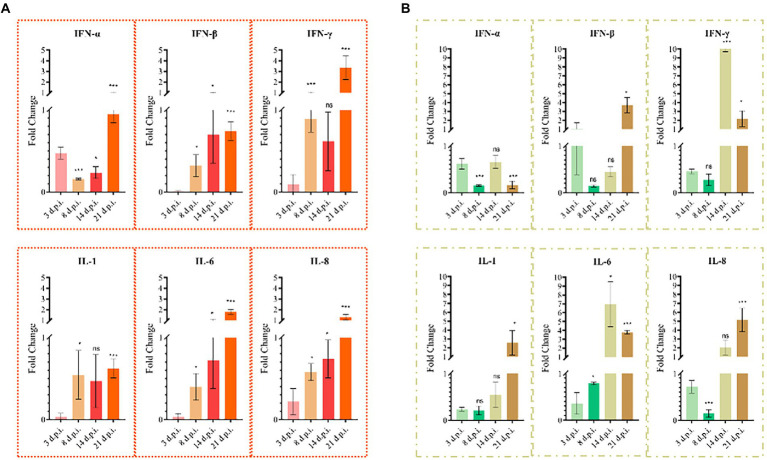
Cytokine changes in chicken thymus and bursa (*n* = 4/group). **(A)** Cytokine changes in the bursa. **(B)** Cytokine changes in the thymus. All columns show average Mean ± SD values.

**Figure 8:**
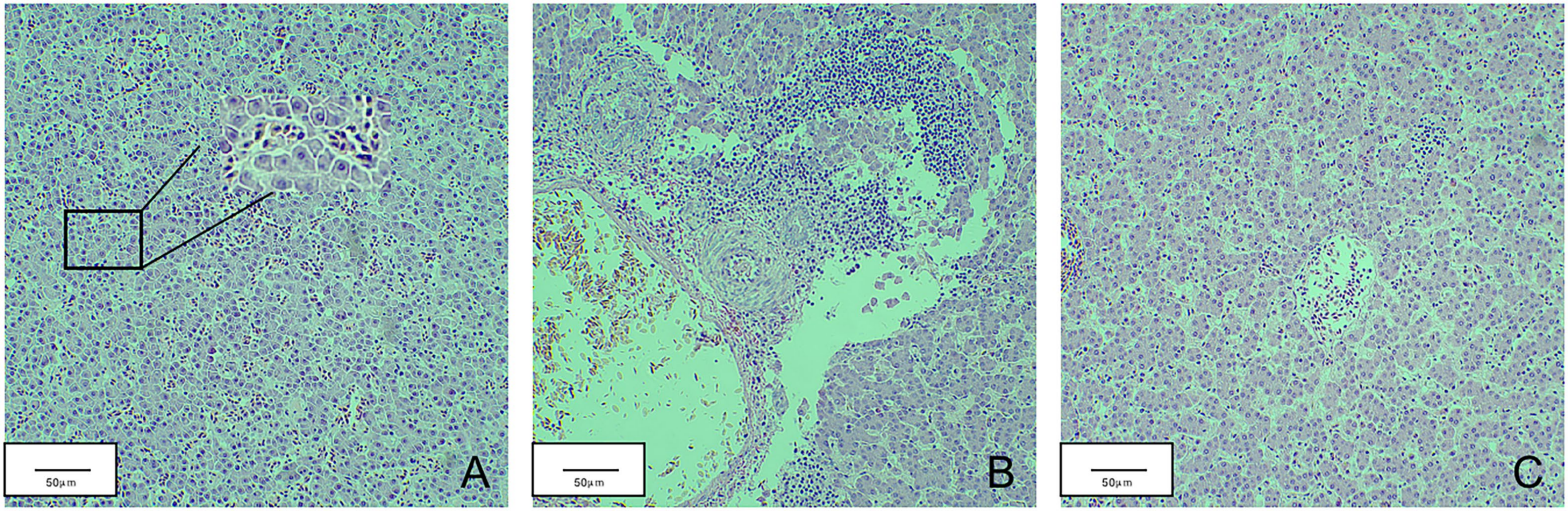
Pathological sections of chicken liver in the JS2020-PFV group. **(A)** Part of the liver spinal cord around the central area of liver disorder. **(B)** Eosinophil infiltration. **(C)** The hepatic sinusoidal space is dilated and congested.

Total RNA was extracted from the thymus and bursa. The expression levels of IL-1, IL-6, IL-8, IFN-α, IFN-β, and IFN-γ were tested using real-time fluorescence quantification PCR. The results revealed that the expression of IL-1, IL-6, IL-8, IFN-β, and IFN-γ first decreased and then increased, whereas IFN-α expression was continuously lower than that of the mock group during detection period (Figure 7). The expression levels of IL-6, IL-8, and IFN-γ of infected chicks were significantly higher than that in the mock group. At 21 d.p.i., the expression levels of IFN-β and IL-1 significantly increased 3–4 times compared with the mock group. However, the expression levels of IL-1, IFN-α, and IFN-β fluctuated during detection and were lower than that of the mock group. At 21 d.p.i., the expression levels of IL-6, IL-8, and IFN-γ were higher than that of the mock group. Livers with obvious lesions were sectioned for pathological examination. The disorder of the hepatic cord around the central liver area, eosinophil infiltration, expansion of the hepatic sinusoidal space, congestion, and edema were observed (Figure 8).

### Detection of CIAV antibody in test chicks

**Figure 9:**
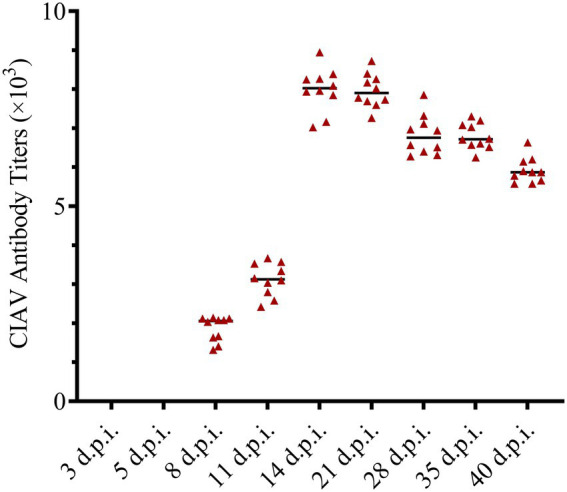
Antibody titers against chicken infectious anemia virus (CIAV) in the JS2020-PFV group. Antibody titers against CIAV = log10 titer =(s/N – 2.72) / (–0.64), s is the A650 of tested sample, N is the A650 of negative control provided by the testing kit.

The anti-CIAV antibody in the JS2020-PFV group and mock group was detected using the IDEXX ELISA kit. The JS2020-PFV group tested positive for the anti-CIAV antibody from 8 d.p.i., and the positive rate continued to rise, reaching 100% at 11 d.p.i. (Table 2). However, the mock group tested negative during the testing period. The titer of the anti-CIAV antibody was converted against the S/N value in the test results. Results showed an upward trend (Figure 9).

### Detection of virus shedding

**Figure 10:**
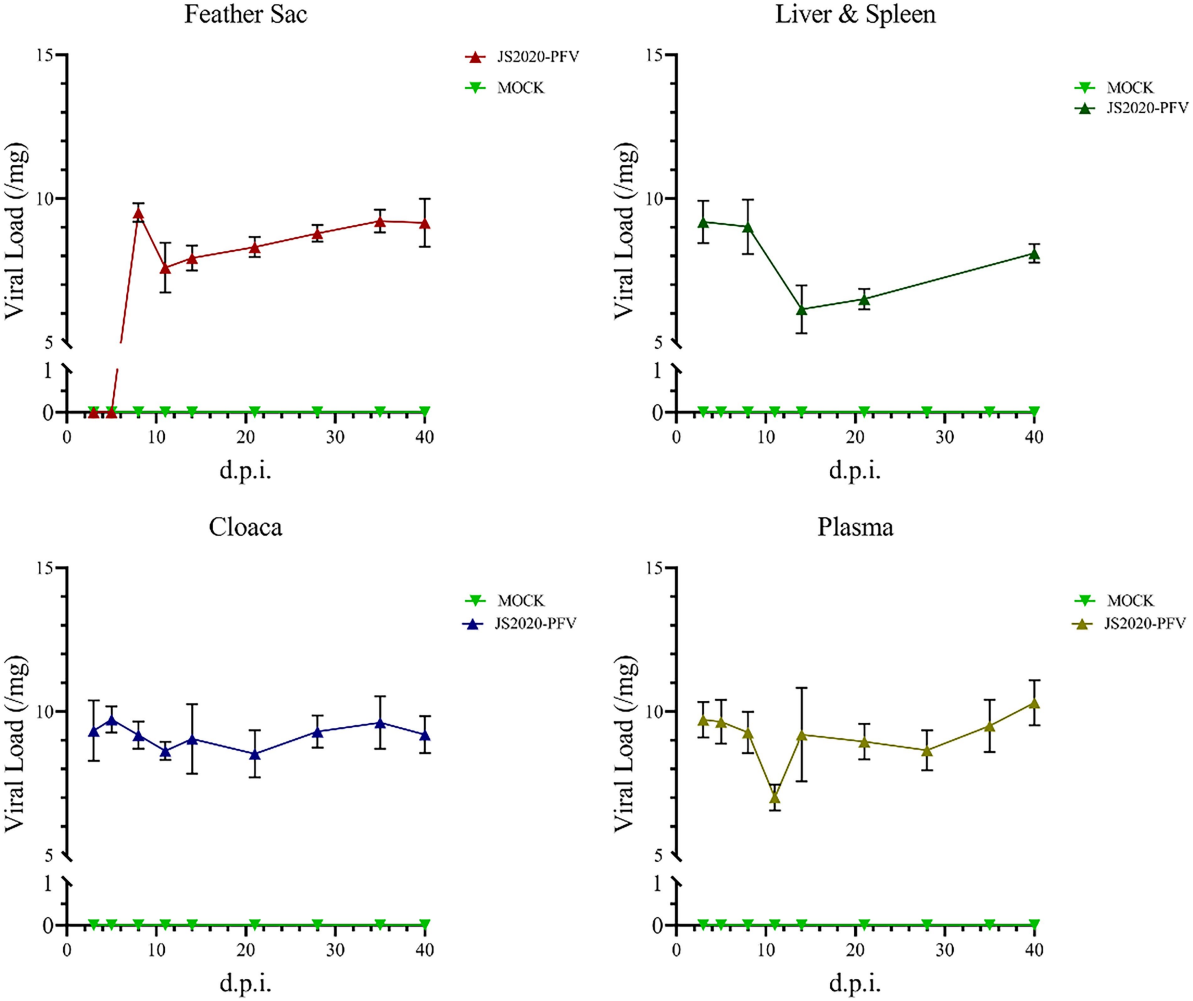
Detection of nucleic acids in the JS2020-PFV group (*n* = 4 per group). Viral Load = log_10_ virus copies. In the JS2020-PFV group, the chicken infectious anemia virus was detected at 8 d.p.i. in the feather sac and at 3 d.p.i in the cloaca, plasma, liver, and spleen. The viral load was relatively low in the liver and spleen.

The liver and spleen of all test chicks were collected after an anatomical examination was conducted at 40 d.p.i., and DNA was extracted and amplified using nested-PCR. A band at approximately 418-bp was observed, thereby confirming that the JS2020-PFV double-copies infection clone was rescued successfully *in vivo* and the CIAV virus effectively replicated in the livers and spleens of SPF chicks. Samples were obtained at different time points and nucleic acid was extracted from them. CIAV infection was detected using the SYBR Green I real-time fluorescence quantification PCR method (Li et al., 2022). The results revealed that CIAV-positive nucleic acid could be detected at 3 d.p.i. in the cloaca, plasma, liver, and spleen and 8 d.p.i. in the feather sac in the JS2020-PFV group. The number of viral copies showed that viral load was relatively low in the liver and spleen (Figure 10).

### Isolation of CIAV from the liver and spleen of JS2020-PFV chicks

**Figure 11:**
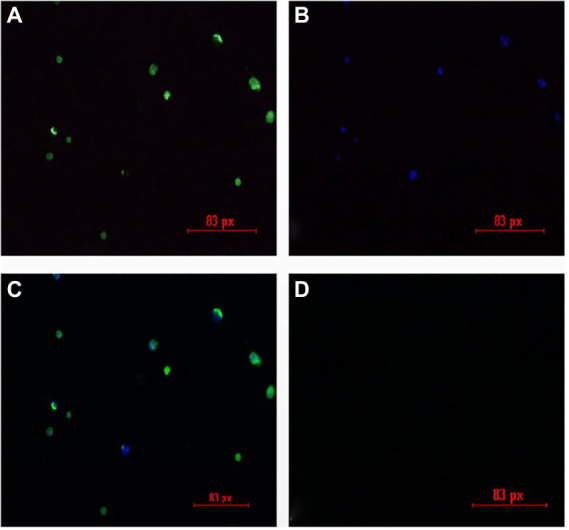
IFA results for the isolation of the chicken infectious anemia virus. **(A)** Positive cells are stained with FITC. **(B)** Positive cells are stained with DAPI. **(C)** Merge plot of FITC and DAPI. **(D)** Negative cells show no fluorescence.

The liver and spleen in the JS2020-PFV group at 40 d.p.i. was multispot sampled and grinded. The fluid in the samples were grinded and used to inoculate MDCC-MSB1 cells. The cells were incubated for 7 days, mixed cultures were tested using IFA. Fluorescent staining analysis was performed using a murine antibody against CIAV-VP3 as the primary antibody, and FITC-labeled goat antimouse IgG was used as the secondary antibody. DAPI staining was used for nuclear staining. As shown in Figure 11, the inoculated cells were stained with green (cytoplasm) and blue (nuclei), whereas mock cells showed no fluorescent signal. These findings indicate that live CIAV was isolated. Thus, live CIAV infection in chicken organs in chicks belonging to the JS2020-PFV group was confirmed.

### Detection of apoptosis characteristics of VP3

**Figure 12:**
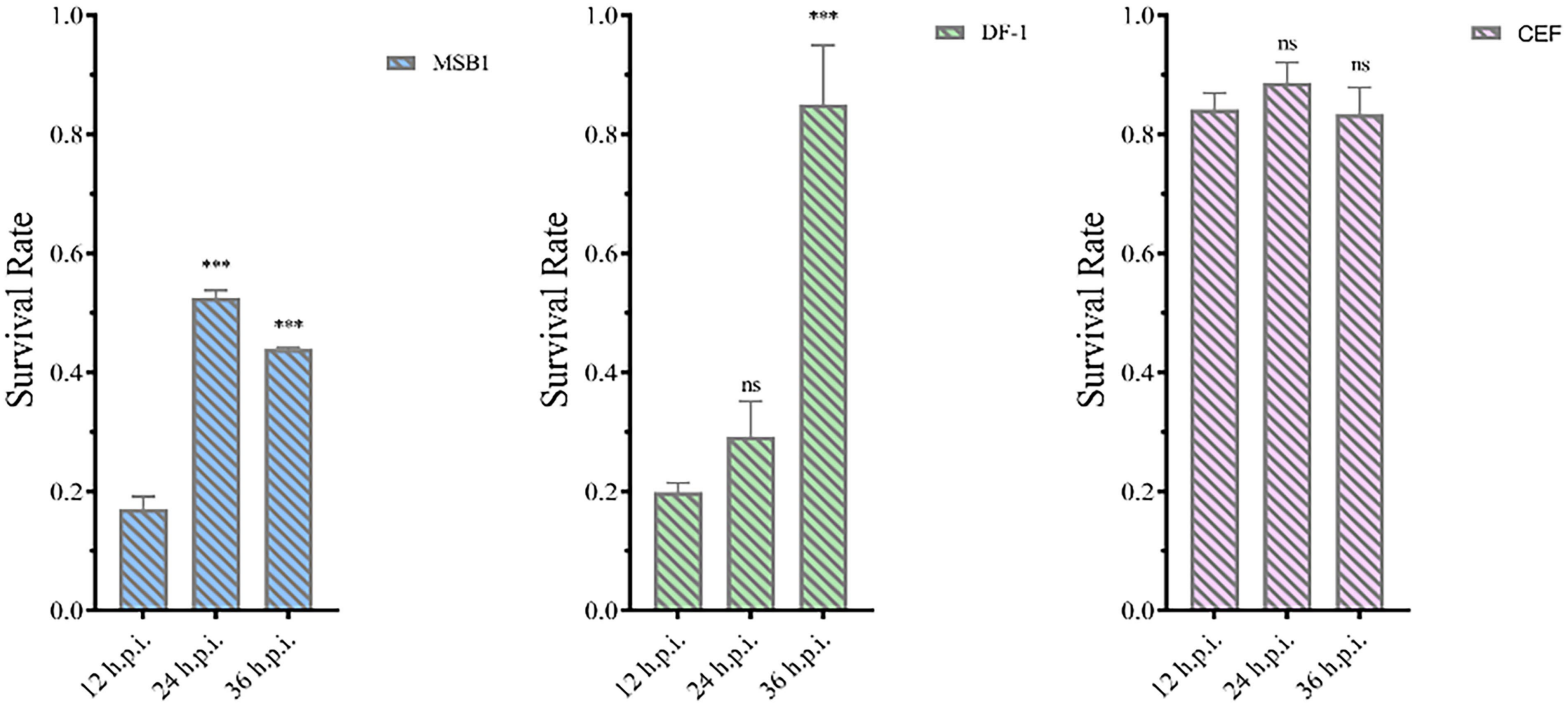
Activity of different types of cells. Activities of MSB1 **(A)**, DF-1 **(B)**, and CEF **(C)** cells at different time points. ***p < 0.001, ns is not significant.

The pET-32a-VP3-CVP1 prokaryotic expression plasmid was transformed into BL-21(DE3) competent cells for expression. The target protein was found in the inclusion body detected using SDS-PAGE gel electrophoresis. The target protein was purified and detected using SDS-PAGE. The target protein was observed at approximately 36 kDa. The protein was quantified at 1183.242 μg/mL. The purified protein solution was inoculated into cells in 6-well plates at 236.648 μg/10^6^ cells. At 12, 24, and 36 h post inoculation, cellular activity was tested using the CCK-8 detection kit. The results showed that although CEF cells were unaffected, MDCC-MSB1 and DF-1 cells showed different degrees of apoptosis, with a significant decline in cellular activity (Figure 12).

## Discussion

Viruses that can be transmitted vertically through chicken embryos, such as ALV (Zavala and Cheng, 2006; Barbosa et al., 2008; Zhao et al., 2014; Wang et al., 2018), REV (Wei et al., 2012; Li et al., 2015), FAdV (Li et al., 2017a,b), and CIAV (Varela et al., 2014; Li et al., 2017a,b), therefore, vaccine produced by SPF chicken embryos contaminated by these viruses can cause exogenous virus contamination. To date, several reports have appeared on the pathogenicity of CIAV in various live avian vaccines, such as the Newcastle vaccine. Unfortunately, there is a lack of research data on contaminated CIAV in live avian vaccines (Su et al., 2018; Huynh et al., 2020). Given that most tests use molecular methods to evaluate the pathogenicity of the isolated CIAV strain, and it is difficult to culture CIAV *in vitro*, constructing the infectious clone is one of the best ways to observe its pathogenicity.

Recently, chickens in a farm showed typical symptoms of anemia after using the attenuated FPV vaccine, indicated the presence of CIAV contaminated in this vaccine. The contaminating strain may have strong pathogenic and immunosuppressive effects.

In this study, the typical symptoms of CIAV infection were observed in SPF chicks after they were inoculated with the JS2020-PFV infectious clone. Infected chicks showed more than 20% mortality, indicating strong virulence of this strain, which is consistent with the prediction of its genomic analysis. The amino acids 75 (V), 89 (K), 19 (K), 125 (I), 139 (K), 141 (Q), 144 (G), 157 (M), and 394 (Q) in JS2020-PFV VP1 correspond with highly pathogenic features. Anemia is the most typical pathogenic manifestation of CIAV (Noteborn et al., 1994). Severe anemia was observed in chicks infected with JS2020-PFV. Typical symptoms of hemorrhage underwing and a lower HCT index was seen in the JS2020-PFV group. JS2020-PFV also had a significant effect on the chicks’ production performance. Chicks in the JS2020-PFV group gained less weight than chicks in the mock group significantly. For example, at 35 d.p.i. the average body weight of chicks in the JS2020-PFV group was only approximately 65% of those in the mock group.

Immunosuppression is one of the typical clinical symptoms of CIAV infection. CIAV infection affects the level of both humoral and cellular immunity to a certain extent. NDV antibody titer of chicks in the JS2020-PFV group was significantly lower than that of the mock group at 22 days post vaccination. When the antibody titer against NDV was 10 in the mock group, it was only about 6 in the infected group. CIAV causes immunosuppression by impeding immune organ development. At different time points post inoculation, the development of the thymus and bursa was significantly inhibited. At 40 d.p.i., when the thymic development index reached 0.81 in the mock group, it was only 0.55 in the JS2020-PFV group, indicating that the thymocyte suffered severe apoptosis after being infected by JS2020-PFV.

CIAV infection can induce apoptosis because its VP3 protein interacts with proteins in the apoptotic signaling pathway (Zhang et al., 2021). VP3 is called apoptin because it can induce apoptosis in chicken thymic lymphoblasts and primitive hematopoietic cells. Wei, et al. found that formation of apoptotic bodies when the VP3 expression plasmid accumulated to a certain value in chicken monocytes. To investigate the apoptotic potential of VP3 of JS2020-PFV, recombinant VP3 protein was inoculated into different cell types to observe its individual effect. Since CIAV VP3 did not freely pass through the cell membrane and needed assistance with cell-penetrating peptides (Li et al., 2012), VP3 was fused with CVP1, which could efficiently penetrate cells (Barka et al., 2004). The activity of different cells was detected after being VP3 inoculation. The results revealed that the primary CEF cells were not affected by the apoptotic effect of VP3 (Jeurissen et al., 1992). However, the activity of the transformed cells, DF-1 and MDCC-MSB1 decreased. This is consistent with studies that have reported that apoptin is distributed in the cytoplasm in normal cells and accumulates in the nucleus of transformed cells to induce apoptosis (Danen et al., 1997). The apoptotic ability of apoptin may be one of the important mechanisms of thymus atrophy induced by JS2020-PFV infection.

In this study, the pathogenicity of the contaminated CIAV strain was systematically researched by constructing a double-copies infectious clone. The pathogenic and immunosuppressive effect of JS2020-PFV on chicks was observed. The findings revealed that the dangers of CIAV contamination in the vaccine cannot be ignored and need to be monitored continuously. It has been proved (Zhuang et al., 1995) that the apoptotic activity of apoptin is not limited to chicken thymocytes, but can also cause programmed death in various human tumors and transformed cells. Thus, fusing VP3 with CVP1 to express the protein can provide a technological roadmap for human tumor treatment.

## Data availability statement

The datasets presented in this study can be found in online repositories. The names of the repository/repositories and accession number(s) can be found in the article/supplementary material.

## Ethics statement

Animal study was reviewed and approved by Shandong Agricultural University Animal care and use Committee (SDAUA-2016-002).

## Author contributions

JW, YL, and LC: methodology and validation. JW, YZ, and YW: formal analysis, data curation, writing—original draft preparation, and visualization. JW, YW, and PZ: writing, review and editing and supervision. PZ, LF, and SC: project administration and funding acquisition. All authors have read and agreed to the published version of the manuscript.

## Funding

This study was funded by the National Key Research and Development Program of China (grant no. 2018YFD0500106) and National Natural Science Foundation of China (grant no. 32102667).

## Conflict of interest

The authors declare that the research was conducted in the absence of any commercial or financial relationships that could be construed as a potential conflict of interest.

## Publisher’s note

All claims expressed in this article are solely those of the authors and do not necessarily represent those of their affiliated organizations, or those of the publisher, the editors and the reviewers. Any product that may be evaluated in this article, or claim that may be made by its manufacturer, is not guaranteed or endorsed by the publisher.
